# Decoding the microbial universe with metagenomics: a brief insight

**DOI:** 10.3389/fgene.2023.1119740

**Published:** 2023-04-24

**Authors:** Hiral G. Chaudhari, Shobha Prajapati, Zuhour Hussein Wardah, Gopal Raol, Vimalkumar Prajapati, Rajesh Patel, Ali A. Shati, Mohammad Y. Alfaifi, Serag Eldin I. Elbehairi, R. Z. Sayyed

**Affiliations:** ^1^ Shri Alpesh N. Patel PG Institute of Science and Research, Sardar Patel University, Anand, Gujarat, India; ^2^ Department of Biosciences, Veer Narmad South Gujarat University, Surat, Gujarat, India; ^3^ Shri R. P. Arts, Shri K.B. Commerce, and Smt. BCJ Science College, Khambhat, Gujarat, India; ^4^Division of Microbial and Environmental Biotechnology, Aspee Shakilam Biotechnology Institute, Navsari Agricultural University, Surat, Gujarat, India; ^5^ Biology Department, Faculty of Science, King Khalid University, Abha, Saudi Arabia; ^6^Department of Microbiology, PSGVP Mandal's S I Patil Arts, G B Patel Science and STKV Sangh Commerce College, Shahada, India

**Keywords:** NGS, next-generation sequencing, metagenomics, microbial diversity, microbiome, shotgun sequencing, amplicon sequencing

## Abstract

A major part of any biological system on earth involves microorganisms, of which the majority are yet to be cultured. The conventional methods of culturing microbes have given fruitful outcomes yet have limitations. The curiosity for better understanding has led to the development of culture-independent molecular methods that help push aside the roadblocks of earlier methods. Metagenomics unifies the scientific community in search of a better understanding of the functioning of the ecosystem and its component organisms. This approach has opened a new paradigm in advanced research. It has brought to light the vast diversity and novelty among microbial communities and their genomes. This review focuses on the development of this field over time, the techniques and analysis of data generated through sequencing platforms, and its prominent interpretation and representation.

## 1 Introduction

**FIGURE 1:**
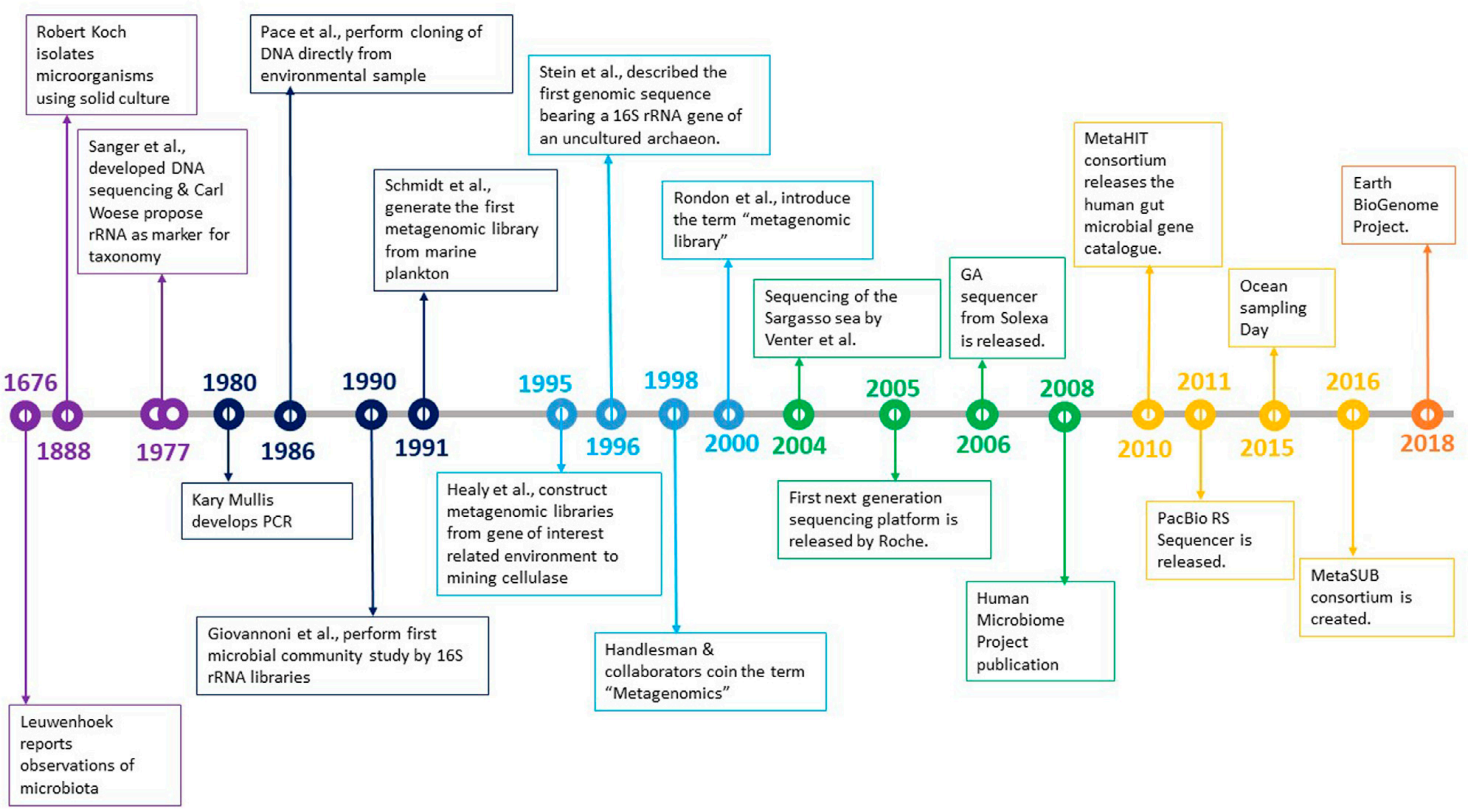
Development in the field of microbiology over the years.

The history of microbiology shows that traditional methods of culturing microorganisms in pure culture fell short of deriving vital information. The time taken to acknowledge the uncultured spectrum was much longer, but certain discoveries helped rekindle the spark to explore that world (Figure 1).

Metagenomics is a technique used for the culture-independent analysis of metagenome, a collective genome representing a whole community (virus, bacteria, or eukaryotic organisms) associated with a particular niche in an ecosystem (Handelsman, 2004; Wajid et al., 2022). The term was first coined by Handelsman (2004). This concept transcends the level of the individual organism to concentrate on the genes in the community and how they can impact one another’s activities in providing collective roles and understanding biology at the aggregate level. Metagenomics also acknowledges the necessity for creating computational techniques that maximize knowledge of community genetics and complex behaviors. It is a combination of genomics, bioinformatics, and systems biology.

Whole-genome shotgun (WGS) and marker gene sequencing have been widely used to examine microbial diversity. WGS sequencing enables the characterization of complete genomes, genes, and genetic characteristics. On the other hand, marker gene analysis offers a detailed account of the variety of specific taxonomic groupings. These methods generate millions of readings, even in a single investigation. As a result, numerous techniques and software have been created concurrently to extract helpful information from the enormous amount of data collected. The structure and content of each microbial community differ significantly, making it challenging to choose the best methodology for evaluating and interpreting such data. Choosing methods, software, and databases compatible with the study’s data and objectives is challenging in this sector. Párez- Cobas and others have reviewed the primary workflows and software used for both approaches and discussed the current challenges in the field (Pérez-Cobas et al., 2020).

Low contigs of genomes are generated and grouped based on the genome they belong to. This process of grouping is called binning. Currently, high-quality metagenome-assembled genomes (MAGs) are developed with binning. This has helped identify thousands of novel organisms and characterize/discover taxonomic and functional components of the microorganisms residing in a particular environment, i.e., microbiomes. These unidentified taxa may be significant to various functions in the biome (Pérez-Cobas et al., 2020).

The meta-DNA is directly extracted from the sample and subjected to sequencing with Next-Generation Sequencing (NGS) platforms. It gives comprehensive information about total microbial diversity and its relative abundance in an ecosystem (Ghurye et al., 2016). The richness and composition of taxonomic groupings present in an environmental sample are revealed by marker gene analyses, which are based on the sequencing of a gene-specific area. The 16S rRNA gene (to analyze the presence of archaea and bacteria), the internal transcribed spacer (ITS) region (to identify the composition of the fungal community), and the 18S rRNA gene are the three main marker genes used in microbial ecology (to report the occurrence of prokaryotic and eukaryotic microorganisms).

Several bioinformatic methods were developed to examine metagenomic data at the molecular (e.g., 16S rRNA), species, and strain levels. The 16S rRNA sequence strategy is one of the most widely used methods for studying microbial taxonomy and phylogeny (Michael and Abbott, 2007; Patel, 2001). Small ribosomal RNA subunit (16S rRNA), the tiny subunit of prokaryotic ribosomes, which is a critical participant in the cellular biology of microorganisms, contains 16S ribonucleic acid acts as a linker for the conversion of genetic material into proteins (Ramazzotti and Bacci, 2018). Sequencing DNA is simpler than sequencing RNA, a DNA segment coding for 16 rRNA is obtained by PCR amplification. This gene fragment satisfies several criteria that have elevated it to the status of a “quasi-gold standard” for bacterial taxonomy: The gene is widespread in the bacteria and archaea domains. 1) It has distinct areas with sufficient variability within its about 1,500 bp to establish a phylogenetic signal among phylum and even genus. 2) It has multiple databases enriched with sequences from practically all international initiatives where 16S sequences are collected. iii) It has conserved sections that enable the design of “universal primers,” a beneficial feature in whole metagenomics.

Metagenomics offers the chance to find new enzymes with industrial uses from extreme habitats where uncultivable extremophiles reside. Functional metagenomics with the help of multiplex techniques allows for the isolation of genes present in a biological sample in the for of proteins or mRNAs, that code for extremozymes, i.e., enzymes that can be catalytically active in harsh conditions or genes that will improve understanding of the mechanisms that make such organisms able to thrive in extreme environmental conditions.

## 2 Steps involved in the workflow of metagenomics

Because of its radically innovative approaches to comprehending the microbial world, metagenomics—still a young science—has already generated a wealth of knowledge about the uncultured microbial world. The first step in every metagenomics study is the direct collection of DNA from every microbe in a certain habitat. After that, the mixed DNA sample can either be directly studied or cloned into a form that can be maintained in lab bacteria, producing a library that contains the genomes of every microorganism in that environment. High-throughput, short-read sequences make up metagenomic samples, and their cost is steadily declining.

### 2.1 The sampling and library construction process

The first and most important stage in every metagenomics research is sample collection and processing. Each type of material must be processed according to specified guidelines, and there are several reliable techniques for the enrichment of genomic DNA (Venter et al., 2004; Burke et al., 2009; Delmont et al., 2011; Thomas et al., 2012). The extracted DNA should be of enough quantity with high-quality nucleic acids and represent all the cells in the sample for subsequent library preparation and sequencing. Typically, this is accomplished by attaching specialized adaptors to one or both ends of the DNA fragments (van Dijk et al., 2014). The DNA adapter will connect the pool of samples to their original sample. Handling DNA at this stage is done with caution to avoid causing chemical, physical, or enzymatic damage to the DNA molecules (Head et al., 2014).

A DNA library is typically constructed using from large DNA fragments (25–200 Kb) collected from environmental sources and cloned into suitable vectors. The size of the insert to be cloned, determines which vector should be used. DNA segments between 100 and 200 kb use cosmids, between 25 and 35 kb use fosmids, between 25 and 40 kb use yeast artificial chromosomes (YAC), and beyond 40 kb are all supported by the bacterial artificial chromosome (BAC). According to the size of their inserts, libraries can be divided into two groups: Small-insert libraries (less than 15 Kb) and large-insert libraries. When compared to large-insert libraries, small-insert libraries have a considerable advantage because the microorganisms can be lysed using harsh techniques that would shear the DNA to an extent that formation of large inserts will not occur (Riesenfeld et al., 2004). Finally, to avoid noise in sequencing data, the free adaptor, adaptor dimers, and any other artifacts need to be eliminated (Head et al., 2014).

### 2.2 Sequencing approaches

Earlier, First-generation sequencing techniques, used chain termination (Sanger and Coulson, 1975) and chemical sequencing technologies (Maxam and Gilbert, 1977). In contrast to the chemical sequencing strategy, the Sanger sequencing method, due to its simplicity and ability to be scaled up, eventually gained widespread application (Schadt et al., 2010). A DNA primer complementary to the template is employed as the starting point for DNA synthesis during Sanger sequencing. The polymerase extends the primer by including the complementary dNTP to the template DNA strand alongwith the four deoxynucleotide triphosphates (dNTPs: A, G, C, and T) that are present. Four dideoxynucleotide triphosphates (ddDNTPs: ddATP, ddGTP, ddCTP, and ddTTP) labelled with different fluorescent dyes are used to stop the synthesis reaction and allow the identification of the nucleotide that is added to the chain of nucleotides. The ribonucleotide in ddNTPs have an oxygen atom removed in comparison to dNTPs, making it incapable of forming a connection with the following nucleotide. Depending on the various chain-terminating nucleotides, the reaction products are inserted into four lanes of a single gel and gel electrophoresis is run. The DNA sequence is thus established based on their size (Sanger et al., 1977). Currently, the use of fluorescent dNTPs in conjunction with capillary electrophoresis offers complete automation of the Sanger method. This change allows for the retrieval of up to 96 sequences each run with an average DNA fragment size of 800–1,000 bp (Venter et al., 2001; Hert et al., 2008; Schadt et al., 2010). Although Sanger sequencing was the foundation of the initial human genome research, it had certain drawbacks. These constraints include high costs and low throughput, among others. It is insufficient for investigating non-cultivable species in complicated ecosystems (Metzker, 2010). DNA sequencing employing reversible terminators was popularly used in the second-generation high-throughput DNA sequencing technology. Dr. Jingyue Ju of Columbia University published the first article on reversible termination sequencing technology (Li et al., 2003). This method’s main distinction from the conventional Sanger sequencing method is that the former method, of second generation sequencing uses modified nucleotide analogous to terminate primer extension reversibly, while the latter, i.e., Sanger sequencing, employs dideoxynucleotide to irreversibly terminate primer extension (Guo et al., 2010).

Recent times have seen a significant increase in the use of several next-generation sequencing (NGS) systems for taxonomic profiling, characterization, and analysis of microbial communities. High-throughput, short-read metagenomic samples are increasingly becoming more affordable as they sequence billions of DNA molecules simultaneously and independently. Also, they do not require the cloning of DNA fragments. The combination of such technologies and metagenomic techniques aided the researchers in studying microbial diversity and understanding the microbial community’s functions and interactions (Shuikan et al., 2020).

## 3 Next-generation sequencing (NGS)

**FIGURE 2:**
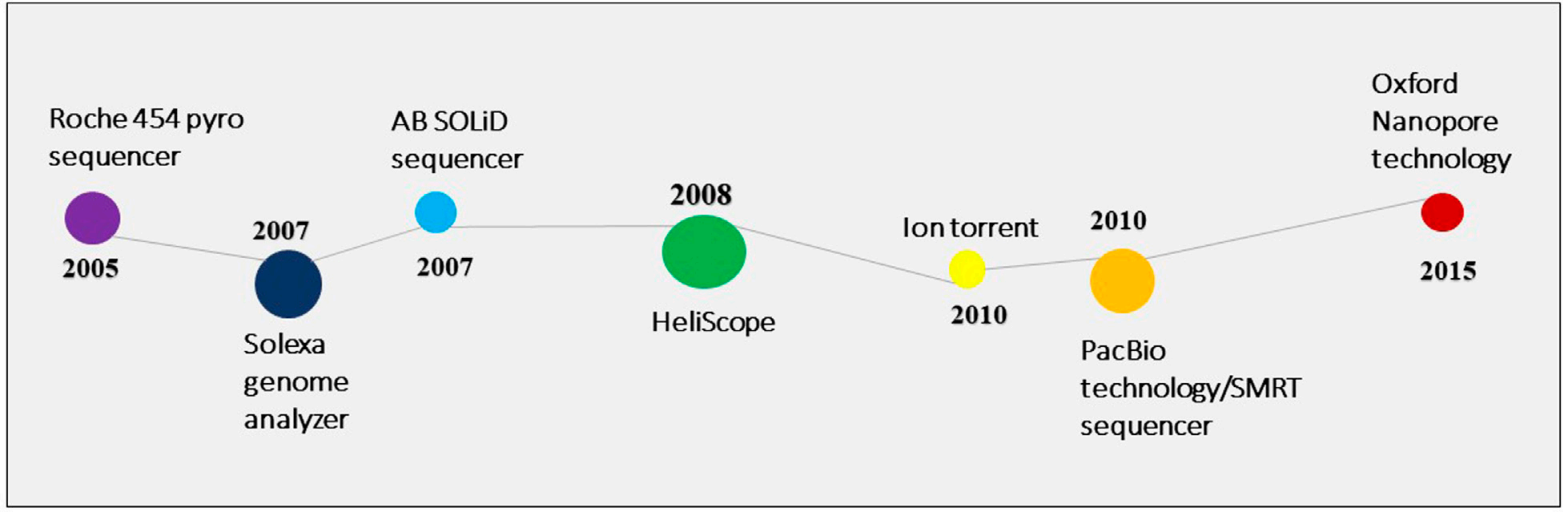
The inventions in the NGS technology.

Owing to the limitations of the Sanger sequencing approach, next-generation sequencing was introduced in 2005 (Varshney et al., 2009). Since 2006, several NGS platforms have been created with numerous applications in the genetic and biological research sectors (Figure 2). A microbial community profile or other information can be assembled from the large volume of data that NGS generates in the form of short reads, much like putting together a puzzle.

The most often utilized platforms are Roche/454 pyrosequencing, Illumina/Solexa sequencing, and Applied Biosystems/SOLiD sequencing. The basis of all NGS is the detection of luminous signals generated by the base incorporation of nucleotides during the sequencing process (Almeida and Martinis, 2018). They also follow the same procedure, which includes DNA extraction, library construction, DNA template preparation, and automated sequence analysis (Vincent et al., 2016).

Next-generation sequencing has enabled without any prior preparation, study, or identification straight from their environments (Sogin et al., 2006). Unlike first-generation sequencing, NGS may simultaneously create hundreds of thousands to millions of sequencing reads. Furthermore, sequencing can be generated without some usual processes, such as vector-based cloning, which decreases the possibility of DNA contamination from other organisms (Mardis, 2008). Some of the NGS platforms are discussed below.

### 3.1 Roche 454 genome sequencer

The real-time sequencing by synthesis (SBS) pyrosequencing method relies on identifying the pyrophosphate (PPi) molecule generated when a nucleotide incorporated into the DNA polymerase is used. The first NGS technique, Roche/454 pyrosequencing, was introduced and made available for purchase in 2005. The 454 Pyrosequencing Technology processes are as follows - The library fragments are joined to beads with oligonucleotides complementary to the adapter sequence ligated at the ends, followed by emulsion PCR to produce DNA beads with millions of copies of the library fragments on their surfaces. The amplified beads are then inserted into a picotiter plate (PTP) with millions of wells. Pyrosequence enzyme beads that have been diluted DNA amplified beads, PPiase beads, and pyrosequence beads are all present in each well. However, only one amplified bead can be held in each. Finally, a CCD camera records the light emission from PTP and converts it to nucleotide sequences (Margulies et al., 2005). Earlier, the longest reading (up to 1,000–1,200 bp) was obtained by 454 pyrosequencing compared to other NGS platforms. Nonetheless, 454 pyrosequencing has the lowest output and cost per base (Huse et al., 2007). Although currently, Pacific Biosciences (PacBio) and Oxford Nanopore Technologies deliver the longest reads (Marx, 2023).

### 3.2 Illumina sequencing (Solexa genome analyzer)

Illumina, originally Solexa, was first commercially available in 2007. Illumina technology employs bridge PCR amplification in conjunction with SBS in the flow cell. The Illumina sequencing premise is that DNA fragments with barcoding primers (adaptors) are linked to the flow cell. In the flow cell, the sequencing procedure is carried out by introducing labeled nucleotides. When the nucleotide is integrated, it produces a luminous signal that optical sensors record. The fluorescent molecules are then withdrawn, and the next tagged nucleotide is incorporated. It uses modified nucleotide analogous to terminate primer extension reversibly during sequencing. Illumina standard sequencing generates a lot of reads (up to 1.5 Tb per run), often with a read length of 150–30 bp and with great precision (error rate ranging from 0.1% to 1%). For instance, the Illumina NovaSeq 6000 System may produce up to 6 Tb per run when used with WGS metagenomics. Given that evaluations of marker gene studies are based on brief fragments (amplicons) and that high quality is required to distinguish between reads, Illumina sequencing is excellent.

Indeed, it is extensively used and popular NGS technology due to its low cost per base and high yield. Illumina sequencing has the large output compared to many NGS, making it appropriate for multiplexing hundreds of samples simultaneously (Glenn, 2011).

### 3.3 Applied Biosystems (AB) SOLiD sequencer

AB SOLiD works on sequencing by oligonucleotide ligation and detection. It was created by Applied Biosystems (Life Technology) and went on the market in 2007. The AB SOLiD sequencing method is distinct from the two major next-generation sequencing technologies, Illumina and 454 pyrosequencing. The AB SOLiD technology uses sequencing-by-oligo-ligation (SBL), while others use sequencing-by-synthesis (SBS). The DNA library is produced from the sample in the SOLiD sequencer, and the appropriate adaptor is subsequently amplified by emPCR (Shao et al., 2011). Instead of DNA polymerase, short nucleotides tagged by DNA ligase are utilized as interrogation probes. The interrogating probe includes six universal bases and a two-base encoded probe. The luminous label is applied to the universal bases. Fluorescent light is created and measured when an integrated probe is ligated with primers using DNA ligase. The subsequent interrogation probe is connected after the 5′ends coupled to the fluorescent label by cleavable linkage are cleaved and removed. This method is performed numerous times until all the targeted DNA is sequenced. SOLiD’s read length is roughly 85 bp, which leads to improper read assembly and requires more time for sequencing, although it has the highest accuracy among other NGS (Liu et al., 2012). SOLiD applications include whole genome sequencing, targeted sequencing, transcriptome, and epigenome analysis (Liu et al., 2012). The drawback of this method is the low data output and its labor intensive.

In addition to the tools already stated, metagenomic research also uses recently developed sequencing technologies that include the single-molecule real-time (SMRT) DNA sequencing from Pacific Biosciences, the Ion Torrent semiconductor sequencing, and the SOLiD 5500 W Series created by Applied Biosystems. More cutting-edge technologies are being developed, which may soon be very helpful for metagenomic research. One of the most exciting new technologies in the genomics era is Irys Technology, created by BioNano Genomics (Oulas et al., 2015). Oxford Nanopore Technologies is actively working on strand sequencing technologies, which allow the sequencing of an intact DNA strand as it travels through a protein nanopore.

### 3.4 Ion torrent sequencing

Life Technology introduced Ion Torrent in 2010. Some writers say the Ion Torrent platform falls somewhere between next-generation and third-generation sequencing techniques. This could be attributable to the approach’s reliance on optical sensors (Liu et al., 2012). It employs the use of a chemical sensor to detect the change in hydrogen-ion concentration that occurs after the insertion of a nucleotide into the sequence rather than fluorescence and camera. The result of sequencing has high quality and is stable (Schadt et al., 2010). Furthermore, compared to pyrosequencing and Illumina (Liu et al., 2012), the Ion Torrent technique is distinguished by its high speed and low cost.

## 4 Third-generation sequencing (TGS) and platforms

The fundamental drawbacks of NGS are the short-read length and PCR bias that occurs due to clonal amplification and fluorescent-based signaling detection (Schadt et al., 2010). As a result, third-generation sequencing, or Single Molecule Sequencing (SMS) methods circumvent these constraints by eliminating PCR before sequencing and capturing the signal in real-time by monitoring the enzyme reaction (Korlach et al., 2010; Schadt et al., 2010). Below we discuss some TGS platforms.

### 4.1 Helicos biosciences (HeliScope)

HeliScope was the first single-molecule-sequencing (SMS) system to be introduced in 2008. It is a fluorescence-based platform for single-molecule sequencing. The preparation stage in the HeliScope platform is based on preparing a single-strand DNA, and there is no requirement for PCR amplification in the preparation step. During sequencing, DNA polymerase and one tagged nucleotide are flowed in repetitive cycles, resulting in DNA template extension dependent on nucleotide flow. The labeled nucleotides are changed by adding a poly-A tail to prevent polymerase extension until a CCD camera captures the fluorescence the inserted nucleotide produces. The unincorporated nucleotides are then washed off, and the fluorescent labels on the strand are chemically erased, allowing for the insertion of the next base (T. D. Harris et al., 2008; Zhang et al., 2011). The HeliScope Genetic Analysis System platform supports RNA sequencing without the requirement for cDNA conversion. Furthermore, due to its short read duration (24–70 bases) and poor data output (20 GB), the HeliScope Genetic Analysis System platform is still in its infancy (Hart et al., 2010).

### 4.2 PacBio technology/SMRT sequencer

In 2010, Pacific Bioscience introduced a single-molecule real-time (SMRT) technology. It is a real-time, fluorescent-based platform for single-molecule sequencing. There is no requirement for PCR amplification during DNA preparation in SMRT (Korlach et al., 2010). A nanostructure known as a zero-mode waveguide (ZMW) is used in this platform for real-time observation of DNA synthesis. A single-stranded template is employed during the sequencing process to manufacture the complementary strand. Unlike other NGS systems, it attaches four different colored fluorescent labels to the terminal phosphate group rather than a nucleotide, releasing a fluorescence signal after nucleotide incorporation (Flusberg et al., 2010). The camera then captures the fluorescent signal in real-time (Timp et al., 2010). The washing step between nucleotide flows is not required in SMRT, which increases nucleotide incorporation and improves sequencing quality (Zhou et al., 2010). SMRT is preferred due to faster sample preparation (hours) compared to NGS, which tends to take days, has no need for PCR amplification during the preparation step, and has a longer read length than any other next-generation sequencing platform (Zhou et al., 2010). Although the quality of MinION and PacBio sequencing is lower than that for Illumina sequences, MinION quality ratings do not correspond to Phred’s predicted error rates (Laver et al., 2015).

### 4.3 Oxford nanopore technology

Oxford Nanopore Technology created nanopore sequencing, which involves passing the DNA sequence through a 1 nm diameter hole (nanopore) where an electric current is applied. The pore’s electrical current is changed for each nucleotide, and the signal is detected in real-time (Hart et al., 2010). Like other third-generation sequencing approaches, this technology does not require PCR amplification or chemical tagging of the sample (Timp et al., 2010). Low-complexity regions are frequently challenging for nanopore sequencers to sequence accurately (minor variation in the electrical signal of the pore when the base does not change). Research from the MinION Analysis and Reference Consortium indicated that the 2D pass readings had a total error of 10.5%, comprising around 3% for mismatch and insertion and slightly more for deletion. DNA translocation speed is not constant, making it challenging to know the exact length. The technology has difficulty determining the difference between the reference genome and sample sequences (Delahaye and Nicolas, 2021). The problem of repetitive sequences, which might affect the metagenome data, was solved by sequencing technologies like PacBio and Oxford Nanopore, but they are still not budget-friendly. Oxford Nanopore Technologies commercialized the MinION in May 2015. The MinION is a pocket-sized portable device that detects bases in real-time (no fluorescent tags), has a long-read duration, and is a low-cost technology (Check Hayden, 2012; Laver et al., 2015; Timp et al.,) collecting samples and sequencing them in the lab, implying that nanopore sequencing will render all other sequencing devices obsolete (Check Hayden, 2012; Jain et al., 2015).

## 5 Metagenomic data analysis

A series of files with the “.fastq” extension comprising the sequence information and the quality for each base are obtained after a sample of sufficient quantity and quality is sent for sequencing. In order to do sequencing quality control, many programmes (including FASTQC and PRINSEQ) use this format, which shows basic data including the total number of bases, read size, GC content, quality for each base on the PHRED33 or PHRED64 scale, and the existence of over-represented sequences (Grabherr et al., 2011; Kornobis et al., 2015; Keegan et al., 2016). The analysed files are passed via a number of tools (Trimmomatic, TrimGalore), which trim the reads of the “.fastq” file depending on the quality of each nucleotide, removing sequences with a PHRED value less than 20, and a minimum fragment size chosen by the user (Kornobis et al., 2015; Keegan et al., 2016). Segments of initiators and sequencing adapters that need to be given in a separate file can be removed using these programmes. These programmes produce archives in the “.fastq” format, which contain common sequences for all samples in a single file and distinct sequences for each sample in a number of files (Kornobis et al., 2015).

For the study of 16S rRNA, several bioinformatics tools are available, including QIIME, MOTHUR, DADA2, UPARSE, and minimum entropy decomposition (MED) (Niu et al., 2018). Some of the more frequently used analysis tools are depicted in Table 1.

**TABLE 1:** Representative software for the NGS data analysis.

Name	Accessibility	Link	References
QIIME	http://qiime.org	http://qiime.org/scripts/index.html#	Caporaso, Kuczynski, et al. (2010a)
QIIME 2	https://qiime2.org	https://docs.qiime2.org/2022.8/interfaces/	Bolyen et al. (2019)
USEARCH	http://www.drive5.com/usearch	https://www.drive5.com/usearch/manual/cmds_all.html	Edgar (2010)
Trimmomatic	http://www.usadellab.org/cms/index.php?page=trimmomatic	http://www.usadellab.org/cms/index.php?page=trimmomatic	Bolger et al. (2014)
Bowtie 2	http://bowtie-bio.sourceforge.net/bowtie2	https://bowtie-bio.sourceforge.net/bowtie2/manual.shtml	Langmead & Salzberg (2012)
MetaPhlAn2	https://bitbucket.org/biobakery/metaphlan2	https://bitbucket.org/biobakery/metaphlan2	Truong et al. (2015)
Kraken 2	https://ccb.jhu.edu/software/kraken2	https://ccb.jhu.edu/software/krakentools/	(D. E. Wood et al., 2019)
MEGAN	https://github.com/husonlab/megan-ce	https://uni-tuebingen.de/fakultaeten/mathematisch-naturwissenschaftliche-fakultaet/fachbereiche/informatik/lehrstuehle/algorithms-in-bioinformatics/software/megan6/	Huson et al. (2016)
	http://www-ab.informatik.uni-tuebingen.de/software/megan6		
MEGAHIT	https://github.com/voutcn/megahit	https://github.com/voutcn/megahit	(D. Li et al., 2015)

The metagenomic composition analyses can be performed using various programs (QIIME, QIIME2, and MOTHUR) that align the reads against a database of ribosomal genes (GreenGenes, SILVA, and RDP) and assign them operational taxonomic units (OTUs) with a distance of 3% and an 80% confidence interval (Schloss et al., 2009; Caporaso et al., 2010a; Caporaso et al., 2010b; Edgar, 2010; Bolyen et al., 2019). Once the OTUs have been assigned, the tools mentioned above calculate diversity indices, richness, and main component analysis, as well as perform sample rarefaction (Babendreier et al., 2007; Schloss et al., 2009; Caporaso et al., 2010a; Caporaso et al., 2010b; Andersen et al., 2013; Bengtsson-Palme et al., 2014; Aires et al., 2015; Apprill, 2017; Afgan et al., 2018; Bolyen et al., 2019). OTU-based methods also have some drawbacks, including the following: 1) OTUs with 97% identity are not always equivalent to species level; 2) due to sequencing errors, the estimated number of OTUs is typically higher than the actual number of species; and 3) OTUs are not sensitive enough to detect minute differences between reads, making it impossible to distinguish between closely related but different taxa. For this reason, non-OTU-based techniques like DADA2 (Callahan et al., 2016), Deblur (Amir et al., 2017), or UNOISE3 (Edgar, 2016) have been created recently to identify precise characteristics known as amplicon sequence variations (ASVs). These tools enable us to examine the diversity of microbes in a variety of habitats, including the water microbiome (Kantor et al., 2019), oral microbiomes (Mukherjee et al., 2018), plant microbiomes (Thompson et al., 2017), and gut microbiomes (Harris et al., 2018; Md Zoqratt et al., 2018; Nearing et al., 2019).

Any one of the inferred single DNA sequences retrieved from a high-throughput investigation of marker genes is referred to as an amplicon sequence variant (ASV). Using ASVs enables the distinction of sequence variation by a single nucleotide change since these analyses, also known as “amplicon reads,” are produced after the removal of incorrect sequences produced during PCR and sequencing. Since ASV-based approaches can identify single-nucleotide variations, they are more applicable than OTU-based methods. ASV estimation techniques can have certain drawbacks, though. For instance, multiple ASVs that differ by more than one nucleotide can exist in a single genome, which could result in incorrect taxonomic annotations. Hence, selecting a more conservative technique, such as the OTU-based methods, is preferable then genomic heterogeneity is crucial to the study (Callahan et al., 2016). Also, the data quality significantly impacts ASV approaches, and PCR mistakes made during the library preparation process result in the loss of a significant amount of usable data. An OTU-based technique is more reliable when the data quality is insufficient (Pérez-Cobas et al., 2020b).

The QIIME software uses visuals and statistics to examine data collected on Illumina or other NGS platforms. Demultiplexing and quality filtering, OTU selection, taxonomic assignment, phylogenetic reconstruction, diversity studies, and visualizations are all part of this (Caporaso, Kuczynski, et al., 2010b; D et al., 2009b). QIIME relies on the PyCogent toolbox to detect misinterpretations and database deposition from raw sequencing results. The lastest versions available are QIIME 18.0 and QIIME2 2023.2 respectively (Caporaso, Kuczynski, et al., 2010b).

The UPARSE software filters and trims read to equal lengths, removes singletons, and clusters the remaining reads. UPARSE can produce operational taxonomic units (OTUs) using NGS data (Edgar, 2013).

MOTHUR, a versatile and comprehensive software program, can evaluate community sequencing data. DOTUR, SONS, TreeClimber, LIBSHUFF, ᴆ-LIBSHUFF, and UniFrac are among the algorithms in the MOTHUR package (D et al., 2009b). DADA2 is appropriate for rectifying amplicon mistakes when there is no possibility of constructing OTUs. To improve the DADA algorithm, DADA2 employs a novel quality-aware model of Illumina amplicon errors (Callahan et al., 2016).

There are at least six metagenomic analysis software options for species-level metagenomic data analysis, including MetaPhlAn2 (Truong et al., 2015), Kraken (Wood and Salzberg, 2014), and MG-RAST (Meyer et al., 2008). All these software tools can be used to profile and score organisms in metagenomic samples. Bowtie2 and UCLUST (Langmead and Salzberg, 2012; Edgar, 2013) are the major algorithms used by MetaPhlAn2, whereas Kraken and CLARK use k-mers (DNA words of length k). For species-level comparisons, MetaPhlAn maps the experimental read against 231 markers, and it maps the reads against >115,000 markers for higher taxonomic levels. This classifier does not require preprocessing, allowing raw data to be uploaded and examined. The fact that MetaPhlAn operates in a Unix architecture through the command line is the primary drawback for non-specialists.

Other taxonomy classifiers are based on comparing previously altered short sequences with single or paired ends (Kraken, Kraken2, OneCodex) with the databases accessible in each application. Kraken uses the Ref-Seq database, where reads are split into fragments known as k-mers and compared to sequenced genomes (Afgan et al., 2018; Wood and Salzberg, 2014). These programs’ output files are in tabular format (tsv), making them easy to export and process in other programs like Vegan or R, where richness, diversity, and rarefaction analyses can be conducted (Conesa et al., 2005; Engel and Moran, 2013; Bengtsson-Palme et al., 2014; Estrada-Peña et al., 2016; Afgan et al., 2018).

Ubiquitous microbial phyla prevalent in samples from arctic, temperate, and tropical habitats, such as Proteobacteria, Actinobacteria, and Cyanobacteria, which are called cosmopolite phyla, have been determined using different taxonomic binning methods. The proportion of each taxon differs between sites, reflecting the circumstances of each ecosystem (Jackson et al., 2009; Truu et al., 2009; Shi et al., 2010; Grabherr et al., 2011; Kanokratana et al., 2011; Cornejo-Granados et al., 2018). A similar pattern has been observed when analyzing the microbiome in other animal models, with the phyla Proteobacteria, Actinobacteria, Firmicutes, and Bacteroidetes being among those with the highest relative abundance (Cornejo-Granados et al., 2018; Cui et al., 2017; Jackson et al., 2009; Kilian et al., 2016; Li et al., 2018; Porchas-Cornejo et al., 2017; Tzeng et al., 2015; Vargas-Albores et al., 2017). This demonstrates that microbial communities are very dynamic, with physical-chemical site variables, health state, and nutrition shaping the metagenome and determining how responsive a microbial community is to environmental changes.

The use of genomic tools has enabled the identification of different organisms’ core microbiomes, given that, despite living in different habitats, they share similar bacterial communities, implying the existence of biological filters that shape bacterium-host interactions, resulting in a stable relationship with the holobiont (Hamdi et al., 2011; Crotti et al., 2012; Aires et al., 2015; Tzeng et al., 2015; Vargas-Albores et al., 2017).

The PICRUST tool was used to do a functional study of the microbial populations. This tool predicts the gene families contained in a metagenome by comparing sequences of gene families previously reported in databases. These predictions are derived in advance for genes that code for proteins found in orthologous gene families (COG) or the Kyoto Encyclopedia of Genes and Genomes (KEEG) (Langille et al., 2013). The STAMP software, which allows for statistical analysis, size effect, and sample corrections, could be used to test the differential expression of these hypothesized functions (Parks et al., 2014). The employment of the techniques above enabled the observation of many characteristics in environmental samples linked to carbon fixation, amino acid metabolism, and signal transduction in lakes, swamps, and other bodies of water (Frias-Lopez et al., 2008; Jackson et al., 2009; Kanokratana et al., 2011; Kornobis et al., 2015; Koo et al., 2017; Porchas-Cornejo et al., 2017). These investigations also revealed the existence of numerous bacterial taxa (Actinobacteria, Verrucomicrobia, and Proteobacteria) capable of synthesizing extracellular enzymes that digest organic materials or mineralize other nutrients (Frias-Lopez et al., 2008; Jackson et al., 2009; Keegan et al., 2016).

The bioinformatics tools mentioned, although open source, needs the user to have specific requirements like the availability of UNIX or OSx operating systems along with a RAM of 16 GB and Hard disk with high storage of more than 500 GB and command lines knowledge (Enrique Valle-Gough et al., 2020). Since these specifications might be challenging to fulfill, there is the availability of Metaserves, which bypasses these shortcomings and provide a graphical environment for data processing.

## 6 Metaservers used for metagenomic studies

Metaservers are online service providers that aggregate disparate programs and applications. Galaxy and MG-RAST are among the most popular meta servers (Kornobis et al., 2015; Keegan et al., 2016; Afgan et al., 2018).

Galaxy is a collaborative project that offers a free set of tools and bioinformatics programs, such as FASTQC for quality control of sequences, sequence editors, data grouping tools, Trinity for tools for assembly, Bowtie for sequence mapping, Salmon and Kallisto for transcript quantification, and Mothur, Vegan, Kraken, and Krona for metagenomic analysis (Shao et al., 2011). Being an open endeavor, Galaxy offers several servers that provide various applications, including servers for the functional annotation of transcripts and the functional prediction of a metagenome by PICRUST (Langille Lab and Huttentowe Lab) (Enrique Valle-Gough et al., 2020).

A free platform called MG-RAST, which stands for Metagenomic Rapid Annotation based on Subsystems Technology (Venter et al., 2001), can analyze sequences from several NGS platforms (Illumina, PacBio, and Nanopore). In contrast to the servers listed above, MG-RAST provides a workflow that comprises sequence quality control, adaptor removal, transcript isoform discovery, taxonomic comparison, and functional assignment. This site offers a number of databases where the findings can be examined in terms of taxonomy (ITS, SILVA, RDP, and GreenGenes) and function (SEED, KEEG, COG, and NOG). Additionally, it offers capabilities for exporting data in fasta, tabular format, or as a matrix of the BIOM type.

## 7 Visualization of sequences data fit by statistical perception

The output and visual representation of the analyzed sequence data is a particularly important aspects of the analysis pipeline. Visualization of data helps us to understand the minor-major difference in the taxonomic composition, its abundance profile relating to alpha and beta diversity, identification of biomarkers, correlation, or network analysis.

Alpha diversity is defined as the diversity within a sample, including species richness and evenness estimation. The most frequently used tools for this kind of analysis include R package, QIIME, and UEARCH. At the same time, the differences between groups in alpha diversity can be statistically calculated using the Analysis of Variance (ANOVA), Mann-Whitney *U* test, or Kruskal–Wallis test (Liu et al., 2019). The beta diversity is defined as the ratio between gamma (regional) and alpha (local) diversities, it is about measuring the differences in samples by relating it with dimensional reduction methods like Principal coordinate analysis (PCoA), constrained principal coordinate analysis (CPCoA) or non-metric multidimensional scaling (NMDS) for visual representation in the form of scatter plots using R vegan package (Oksanen et al., 2020).

Taxonomic composition is visualized using the stacked chart, bar plot, *etc.*, where microorganisms’ richness is presented at the phylum or genus level with its respective percentage abundance level in the plot. Correlation analysis has shown the relation between taxa and sample metadata, including details like environmental factors such as pH, latitude, longitude, temperature, and others (Edwards et al., 2018). There are various ways and methods to represent the analyzed metagenomic data for ease of understanding and appropriate interpretation, as shown in Table 2. To increase the reproducibility of the performed analysis, the data file, metadata, and code must be submitted along with the publication script. The researchers can upload raw data with its metadata file in any data repository like the National Centre for Biotechnology Information (NCBI), European Bioinformatics Institute (EBI), Genome Sequence Archive (GSA) by Beijing Institute of Genomics Chinese Academy of Sciences, *etc.* Researchers can also share and accommodate the analysis pipeline script, running environment, and its version to a detailed statistical and visualization report which is significantly effective for other researchers or the scientific community to get it to reproduce as supplementary material (Y. Liu et al., 2019).

**TABLE 2:** The different analyses of the sequence data and its visualization methods.

Method	Visualization	
Alpha diversity	Boxplot	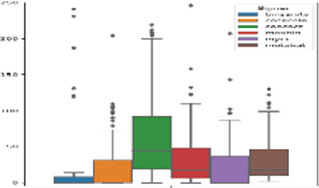
	Rarefaction curve	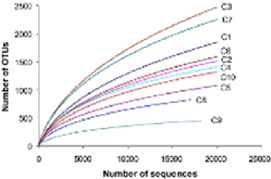
	Venn diagram	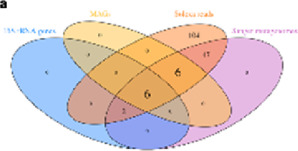
Beta diversity	Unconstrained PCoA scatter plot	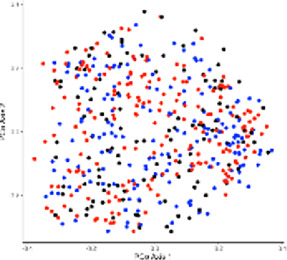
	Constrained PCoA scatter plot	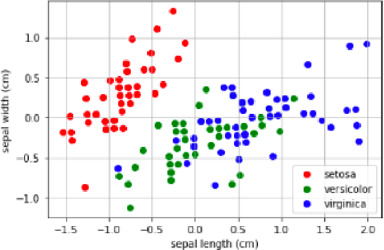
	Dendrogram	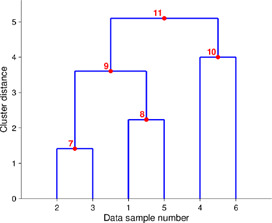
Taxonomic composition	Stacked bar plot	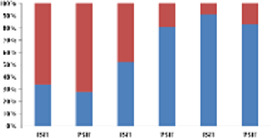
	Flow or alluvial diagram	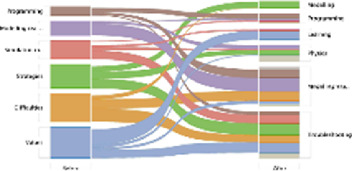
	Skanky diagram	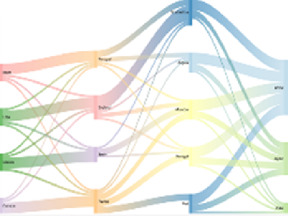

## 8 Applications

**FIGURE 3:**
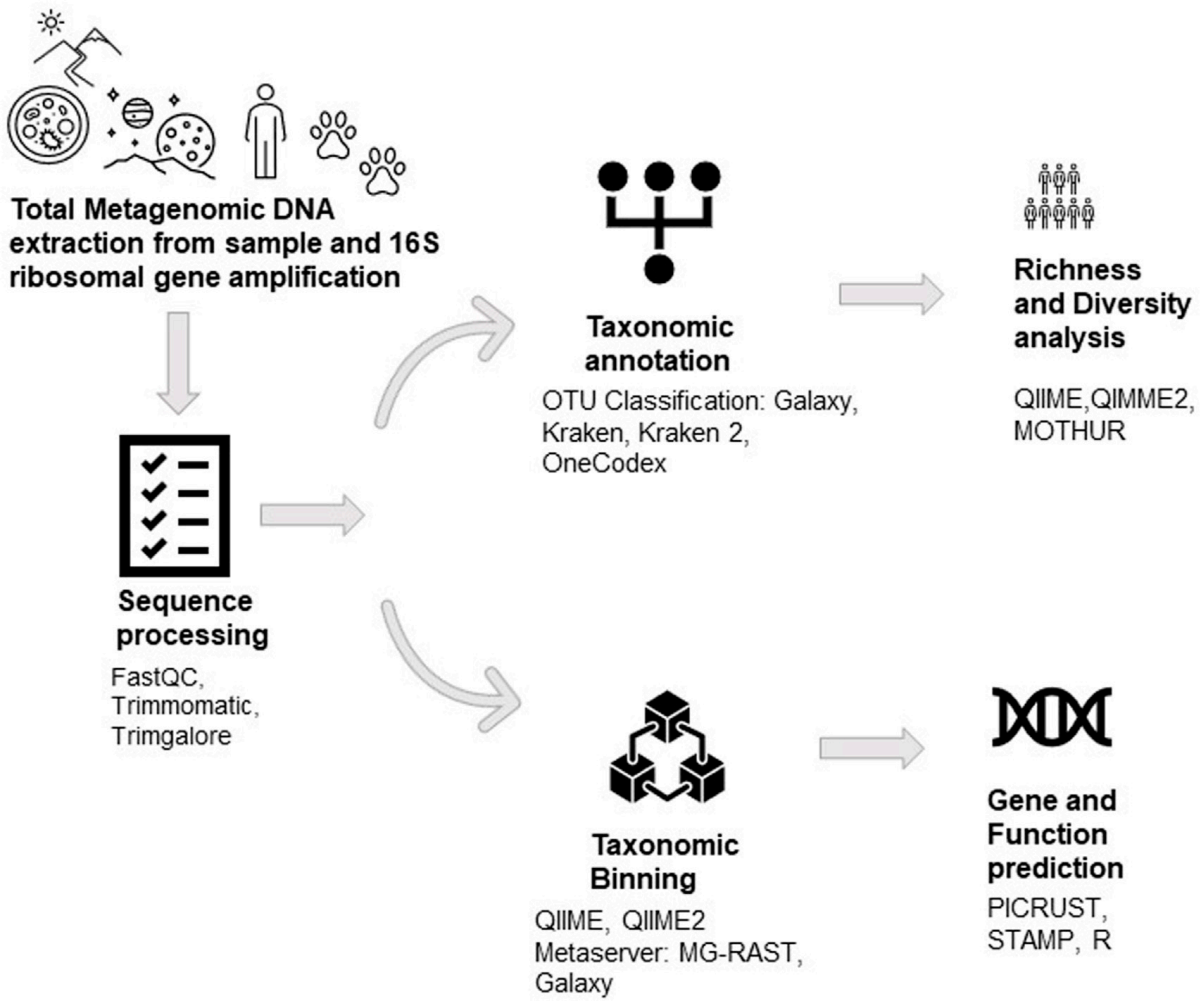
The workflow of Metagenomic DNA analysis.

At the outset, scientists around the globe were trying to dig out novel biomolecules through conventional methods. Still, it seemed almost impossible and was later answered by the metagenomes of any ecological niches. Understanding and evaluating the metagenome can only be possible with the advent of Next-Generation Sequencing techniques which enables researchers to hypothesize studies on new concepts like microbial community dynamics, comparative community study, meta-transcriptomics, and meta-proteomics, *etc.* Through the investigation of 16S ribosomal sequences, genomic techniques have revealed extraordinary variety and bacterial ubiquity in several types of samples in recent years (Babendreier et al., 2007; Shi et al., 2010; Hamdi et al., 2011; Andersen et al., 2013; McFall-Ngai et al., 2013; Aires et al., 2015; Kornobis et al., 2015; Keegan et al., 2016; Papkou et al., 2016; Apprill, 2017; Xiong et al., 2018) (Figure 3). These techniques have enabled the molecular investigation of populations and the establishment, regulation, and evolution of several biological processes (Andersen et al., 2013; D et al., 2009a; Hamdi et al., 2011).

Metagenomics has been used to study the microbial community of varied environments, including extreme environments. It is so unique that it is impossible to mimic strictly at the laboratory level. The microbial community composition of hypersaline environments like Lake Meyghan (Naghoni et al., 2017), lithium-rich Salar de Uyuni (Haferburg et al., 2017; Vera-Gargallo and Ventosa, 2018), The desert of Kutch (Pandit et al., 2015), Karak Salt mines (Cycil et al., 2020), Aquatic hypersaline environments (Ghai et al., 2011), Freshwater lakes of Amazon Basin (Toyama et al., 2016), Phumdi at Loktak Lake (Puranik et al., 2016), Hot water springs (Ghelani et al., 2015; Mangrola et al., 2015; Wilkins et al., 2019), Solar salterns (Manikandan et al., 2009), *etc.*, have been evaluated in depth because of the metagenomics.

Besides being used for community analysis, the approach has also been used to mine enzymes (Robinson et al., 2021). Industrially relevant and very important enzymes have been discovered using metagenomics, which includes oxidoreductases, glycosyl hydrolases, proteases, lipases, and phosphatases (Wang et al., 2004; Berini et al., 2017; Prayogo et al., 2020; Verma et al., 2021). Many of them have already been patented, like xylanase (Patent no. EP 2990482 A1) sourced from a hot spring which can be used in biofuel production from lignocellulosic biomass, β-galactosidase (Patent no. EP 2530148 A1) which can be used in the food processing industry, Esterase (US20160053239 A1) from brine pool can be used in leather manufacturing or oil biodegradation. Some of them have been commercialized include, Luminase of class Xylanase, which is used in paper production, and Phyzyme XP, a Phytase used as an additive in livestock feed (Berini et al., 2017). Patel et al. explored metagenomic research on enzyme exploration for lipase, protease, and cellulase of microbial origin by enzyme mining through metagenomics (Patel et al., 2022).

The influence of the microbiome on host function has been proposed as a co-evolutionary process in which the functionality and composition of the microbiome can be influenced by the host’s feeding habits (Frias-Lopez et al., 2008; Kilian et al., 2016) and the host can benefit from specialized microorganisms capable of synthesizing metabolites that were not originally present in the environment (Shi et al., 2010; Cornejo-Granados et al., 2018). Japanese seaweed intake allows the entrance of algae-associated bacteria, which transmit the genes involved in the breakdown of algal sulphated polysaccharides to competent gut resident bacteria via a process known as horizontal gene transfer (Hamdi et al., 2011). Certain sea invertebrates (Elysia chlorotica) that feed on algae can keep the algal plastids as photosynthetic symbionts, allowing them to eat photosynthates (McFall-Ngai et al., 2013). These coevolutionary processes demonstrate how the host’s food habits can alter the functionality of the microbiome because these metabolic add-ons allow the host to flourish in otherwise harsh environmental situations (oligotrophic habitats).

Further, the identification of different organisms’ core microbiomes, given that, despite living in different habitats, they share similar bacterial communities, implies the existence of biological filters that shape bacterium-host interactions, resulting in a stable relationship with the holobiont. Recent research in farm animal faeces has revealed the presence of host-specific intervening sequences (IVS), which offer a foundation for distinguishing microbes from different hosts (Shen, 2016). In the case of *A. mellifera*, a global core microbiome composed of Proteobacteria, Firmicutes, Bacteroidetes, and Actinobacteria has been identified, as well as a high concentration of lactic acid bacteria, which have a beneficial activity in the host organism’s health due to their involvement in the immunomodulation of the intestinal microbiota (Babendreier et al., 2007; Cornejo-Granados et al., 2018; Mohr and Tebbe, 2006). The presence of symbiotic microorganisms within the intestinal tract of various animal species (*A. mellifera, Litopenaeus vannamei, Mus musculus*, and *Homo sapiens*) has been demonstrated to be important for survival, as cooperative behavior boosts the strength of a community (Cornejo-Granados et al., 2018; Crotti et al., 2012; Hamdi et al., 2011; Macfarlane and Dillon, 2007; Turnbaugh et al., 2009).

The importance of microbial communities within a host cannot be overstated. Given the delicate balance of these interactions, any changes in the microbiome makeup could cause disease in the host (Shi et al., 2010; Tzeng et al., 2015; Estrada-Peña et al., 2016; Cornejo-Granados et al., 2018). The presence of pathogens in marine settings has been documented in ambient samples (Frias-Lopez et al., 2008; Porchas-Cornejo et al., 2017) as well as in numerous marine organisms (*L. vannamei* and *M. nipponense*) (Tzeng et al., 2015; Vargas-Albores et al., 2017; Cornejo-Granados et al., 2018; Xiong et al., 2018). Metagenomics and its approaches are widely used in various fields, which has opened a floodgate of knowledge of the microbial world and how it functions.

## 9 The multiomics

Multi-omics (multiple omics) offers an integrated approach to drive biological discovery at several levels. To quantify gene expression, gene activation, and protein levels, this biological analytic method combines genomic data with information from other modalities like transcriptomics, epigenetics, and proteomics.

Current trends focus on metatranscriptomics, which entails sequencing the entire (meta) transcriptome of the microbial community, which is a better method for determining a microbial community’s functional profile than whole-metagenome sequencing (WMS), which only gives a partial view. We can learn about the genes expressed by the community as a whole by metatranscriptomics.

It is feasible to deduce the functional profile of a community under particular circumstances, which are typically reliant on the status of the host, by using functional annotations of expressed genes. While metatranscriptomics and metagenomics contribute to the understanding of the questions “what genes are collectively expressed under different conditions?” and “what is the composition of a microbial community under different conditions?” metabolomics focuses on the question “what byproducts are produced under different conditions?” (Aguiar-Pulido et al., 2016).

With new tools (MetaVelvet, TriMetAss, and MetaAmos) specifically created to get full genomes and transcriptomes of the bacterial communities, current research is being carried out to achieve more exact metagenomic and metatranscriptomic assemblages. The methods like meta-transcriptomics and meta-proteomics are promising approaches which will help us study gene expression, proteins profiles and their roles in biological cycles and their response to the environmental factors which may govern their functioning. These methods could aid in a better understanding of the intricate metabolic and trophic networks that operate in an organism or environment, along with the integration of other “omic” approaches and systems biology.

The Institute of Physics of Cantabria created the free server TRUFA: Transcriptome User-Friendly Analysis (Kornobis et al., 2015), which includes several programs only for transcriptomic (metatranscriptomic) analysis, including quality control (FASTQC and PRINSEQ), edited of sequences (CutAdapt), assembled of sequences (Trinity), quantification of transcripts (RSEM and eXpress), and functional annotation (BLAST2GO and HMMER). The files can be modified beforehand, and some platform modules can be accessible, such as the functional annotation for previously assembled sequences.

## 10 Conclusion

The field of metagenomics has grown tremendously after the development of high throughput sequencing technologies, Next-Generation Sequencing methods, and Bioinformatics tools, which have helped us to overcome culture bias. Initially, researchers focused on microbes of temperate environments, but now the study and vision has shifted to much challenging inhospitable environments and the microbial community. We urgently need an opportunity to thrive for a long time, hoping to gain something novel within the microbial world to help us understand the mechanisms and interactions of extreme environments and the role of associated microbes. The future application and its implementation depend on how we can develop the process and techniques and make them easily accessible and affordable because of the growing interest in the fraternity of researchers around the globe. The combination of metagenomics, meta-transcriptomics, meta-proteomics, and metabolomics can answer many unresolved questions, solve the microbe’s mystery, and help us analyze the microbial communities and their interactions.

## Abbreviations

ASVs, Amplicon Sequence Variants; GUI, Graphic User Interface, HMP, Human Microbiome Project; ITS, Internal Transcribed Spacers; MAGs, Metagenome Assembled Genomes; NGS, Next-Generation sequencing; OTUs, Operational Taxonomic Units; PE, Paired-End; PTP, picotiter plate; QC, Quality Control; QIIME, Quantitative Insights into Microbial Ecology; RDP, Ribosomal Database Project; SBL, sequencing-by-oligo-ligation; SBS, sequencing by synthesis; SMRT, single-molecule real-time; SMS, Single Molecule Sequencing.
